# Myelin water and tensor‐valued diffusion imaging: (How) are they related?

**DOI:** 10.1002/mrm.30620

**Published:** 2025-07-01

**Authors:** Sharada Balaji, Adam V. Dvorak, Neale Wiley, Erin L. MacMillan, Anthony Traboulsee, Irene M. Vavasour, Guillaume Gilbert, G. R. Wayne Moore, David K. B. Li, Cornelia Laule, Alex L. MacKay, Shannon Kolind

**Affiliations:** ^1^ Physics and Astronomy University of British Columbia Vancouver British Columbia Canada; ^2^ MR Clinical Science, Philips Canada Mississauga Ontario Canada; ^3^ Radiology University of British Columbia Vancouver British Columbia Canada; ^4^ Medicine (Neurology) University of British Columbia Vancouver British Columbia Canada; ^5^ International Collaboration on Repair Discoveries University of British Columbia Vancouver British Columbia Canada; ^6^ Pathology and Laboratory Medicine University of British Columbia Vancouver British Columbia Canada

**Keywords:** b‐tensor diffusion, microscopic fractional anisotropy, myelin water imaging, tensor‐valued diffusion, tract profiling

## Abstract

**Purpose:**

Conventional MRI offers limited insight into specific characteristics of central nervous system tissue, whereas quantitative MRI measures can provide more detailed information about different aspects of microstructure. A multi‐metric approach involving multiple quantitative measures may improve our understanding of healthy tissue and pathology. Previous work shows myelin water fraction (MWF) is related to fractional anisotropy (FA), but this relationship is complicated by confounding factors that may be resolved using tensor‐valued diffusion imaging, which yields measurements of microscopic FA (μFA) and tissue heterogeneity (C_MD_). Our aims were to better understand how measures from myelin water and tensor‐valued diffusion imaging relate to one another, and to demonstrate how these measures can be used to characterize microstructure in both healthy white matter and pathological changes.

**Methods:**

We assessed the relationship between MWF, FA, μFA, and C_MD_ from 25 healthy individuals through atlas comparison, correlation analysis, and tract profiling. We also applied z‐score analysis and tract profiling in five people with multiple sclerosis (MS) to evaluate the multi‐metric utility of these measures in assessing pathology.

**Results:**

Although correlation analysis showed moderate, but potentially misleading relationships between metrics, tract profiling showed consistent tract‐specific pattern differences between metrics in healthy tissue. In MS, MWF, μFA, and C_MD_ were the most sensitive to pathological changes, showing regions of abnormality even in normal‐appearing white matter and along lesional tracts, and highlighting different types of damage.

**Conclusion:**

Using MWF, μFA, and C_MD_ to separately assess myelination, anisotropy, and tissue heterogeneity enhances our ability to investigate development, aging, disease, and injury.

## 1 INTRODUCTION

In vivo characterization of healthy tissue microstructure and microstructural changes that occur because of pathology is crucial for understanding central nervous system (CNS) aging, injury, and disease. It is an ongoing challenge in MRI to distinguish between the various tissue environments that may exist within a typical imaging voxel. In neurological diseases such as multiple sclerosis (MS), different types of microstructural changes can occur simultaneously. For instance, MS lesions may demonstrate inflammation, de‐/re‐myelination, axonal damage, gliosis, and/or edema.^1^ These pathological processes are indistinguishable on conventional MRI. Many advanced MRI methods have been developed with the aim of probing microstructure non‐invasively, but quantitative metrics from different techniques may provide conflicting information or support the same claim without providing unique information.^2^ Using multiple imaging approaches can provide measures specific to different microstructural components within an imaging voxel, such that the role of, interaction between, and changes in, these various tissue components can be better characterized.

Myelin water imaging (MWI) is a histopathologically validated MRI technique to measure myelin content in the CNS.^3^, ^4^, ^5^, ^6^, ^7^, ^8^, ^9^, ^10^ MWI typically involves acquiring multi‐echo data using a Carr‐Purcell‐Meiboom‐Gill^11^ or gradient and spin echo^12^ sequence to produce voxelwise T_2_ decay curves, which can be decomposed into T_2_ distributions using a non‐negative least squares algorithm.^13^ The T_2_ components that exhibit short T_2_ times (<40 ms) are attributed to water trapped within the myelin bilayers, whereas components exhibiting longer T_2_ times (40–200 ms) are attributed to intra‐ and extra‐cellular water (usually not separable). The ratio of the short‐T_2_ myelin water signal to the total water signal gives a quantitative metric called the myelin water fraction (MWF).^13^ MWI was historically hampered by lengthy scan times,^13^ but acquisition methods are now approaching clinical feasibility.^14^ A recent technique to accelerate the acquisition of the T_2_ decay curve using a compressed sensing style acquisition with a constrained subspace reconstruction (CALIPR)^15^ can provide whole‐brain MWI data. Combining CALIPR with a 3D spatial correlation‐based analysis technique leads to MWF maps that are more robust to noise and have improved sensitivity for detecting pathology.^16^

Brain microstructure can also be probed using diffusion imaging approaches such as diffusion tensor imaging (DTI). As DTI assumes each voxel has a single water compartment with a Gaussian diffusion profile, microstructural features like fractional anisotropy (FA), tissue heterogeneity, and fiber orientation dispersion are tangled together in the signal. Models that can quantify different sub‐voxel tissue compartments, such as neurite orientation dispersion and density imaging (NODDI), composite hindered and restricted model of diffusion (CHARMED), and diffusion basis spectrum imaging (DBSI), are based on assumptions of tissue characteristics, for example, constraints on diffusivities; however, such constraints may fail in the presence of pathology.^17^ Tensor‐valued diffusion imaging^18^ can provide a model‐free signal representation and metrics to describe tissue microstructure. Voxels are assumed to be composed of multiple micro‐environments, each with a unique diffusion tensor, and the means and variances of the diffusion tensors within each voxel are represented. From this framework, the microscopic FA (μFA) is proposed to quantify anisotropy with less ambiguity than FA.^18^, ^19^, ^20^ Characterizing tissue heterogeneity separately is also desirable given that pathology can change the relationship between tissue heterogeneity and diffusion anisotropy. C_MD_ ^18^ provides information about the variance of diffusion tensor sizes (i.e., diffusion tensor traces) within a voxel, which relates to tissue heterogeneity within the voxel.

All of the measures under consideration (MWF, FA, μFA, and C_MD_) reflect aspects of brain microstructure. Our aims were to better understand how these measures relate to one another in healthy and MS brain and to demonstrate how these measures can be used to characterize microstructure in both healthy white matter (WM) and pathological changes. We investigated these relationships in 25 healthy participants by: (1) creating atlases of MWF, FA, μFA, and C_MD_ to assess the variability of measures across the healthy population; (2) assessing correlations between the measures across different brain structures to see how they relate at the level of overall regions; and (3) profiling measures along WM tracts (tractometry) to visualize their spatial variation in healthy WM. We then investigated the measures in five example cases of people with MS to demonstrate the heterogeneity of tissue microstructure changes by (4) assessing z‐score maps of MWF, FA, μFA and C_MD_, created based on the healthy atlases; and (5) assessing tract profiles along lesional tracts and comparing with healthy average tract profiles.

Previous studies^21^, ^22^, ^23^, ^24^ have compared MRI measures of myelin content and measures from linear diffusion encoding, finding moderate relationships between MWF and FA with some dependence on orientation dispersion. The novelty of this work lies in its comparison of MWF from the CALIPR framework with measures from tensor‐valued diffusion, and demonstration of the acquisition and analysis techniques in MS.

## 2 METHODS

### 2.1 Participants and data acquisition

MRI data was acquired in 25 healthy controls (HC) and five participants with MS (demographic information in Table 1). All experimental protocols were approved by the University of British Columbia Clinical Research Ethics Board (no. H17‐00866), and all methods were performed in accordance with relevant guidelines and regulations. All volunteer participants provided informed, written consent. Imaging was performed at 3 T (Philips Ingenia Elition X, Philips Healthcare, Best, the Netherlands) using a 32‐channel receive‐only head coil.

**TABLE 1:** Demographic information: Sex, age, EDSS, disease duration, and current treatments.

	Healthy participants	Relapsing–remitting MS	Progressive MS
Sex	11 M/14 F	1 M/1 F	3 F
Age	46 ± 15 y (23–70 y)	62, 62 y	69, 63, 68 y
EDSS	–	2.0, 3.5	3.5, 6.0, 6.5
Disease duration	–	13, 15 y	32, 47, 39 y
Treatments	–	Ocrelizumab, none	Teriflunomide, baclofen, gabapentin

Abbreviation: EDSS, expanded disability status scale; F, female; M, male; MS, multiple sclerosis.

A 3D T_1_‐weighted turbo field echo (TFE/MPRAGE/fast spoiled gradient echo) sequence was acquired (shot interval/TR/TE/TI = 2400/7.7/3.7/950 ms, FOV = 256 × 180 × 256 mm^3^ [anterior‐posterior (AP) × right‐left (RL) × foot‐head (FH)], 1 × 1 × 1 mm^3^, compressed sensing‐sensitivity encoding (CS‐SENSE) factor = 3.6, time = 3.5 min) for anatomical segmentation, registration, and to create a structural atlas. MWI data was collected using the CALIPR sequence^15^ (56 echoes, TR/ΔTE = 1252/6 ms, FOV = 240 × 200 × 100 mm^3^ [AP × RL × FH], acquired at 1.7 × 1.7 × 1.7 mm^3^, reconstructed to 1 × 1 × 1 mm^3^, undersampling acceleration factor = 23.9, time = 7.5 min). Tensor‐valued diffusion imaging acquisitions with spherical tensor encoding (STE, 33 rotations, δ_Before_/δ_After_ = 49.58/45.22 ms, time = 3 min, *b* = 0, 100, 700, 1400, 2000 s/mm^2^), planar tensor encoding (PTE, 32 rotations, δ_Before_/δ_After_ = 49.58/45.22 ms, time = 3 min, *b* = 0, 100, 1000, 2000 s/mm^2^) and linear tensor encoding (LTE, 59 directions, δ_Before_/δ_After_ = 41.5/41.5 ms, time = 5 min, *b* = 0, 100, 700, 1400, 2000 s/mm^2^) were also performed.^25^, ^26^, ^27^ For all tensor‐valued diffusion acquisitions, multiband SENSE factor = 2, SENSE factor = 1.9, half‐scan factor = 0.74, TR/TE/δ_pause_ = 5200/129/17.5 ms, FOV = 240 × 240 × 144 mm^3^ (AP × RL × FH), resolution = 3 × 3 × 3 mm^3^, with δ_pause_ being the time between the end of the first and start of the second diffusion encoding gradient, and δ_Before_/δ_After_ being the diffusion encoding gradient duration before and after the refocusing pulse.^25^, ^26^ Waveforms were generated using an open‐source generator.^27^ For one healthy volunteer, the same tensor‐valued diffusion sequences at resolution = 2.25 × 2.25 × 2.25 mm^3^, FOV = 223 × 240 × 99 mm^3^ (AP × RL × FH) were also acquired to investigate partial volume effects. Reverse phase‐encoded *b* = 0 images were acquired for 24 of 30 participants. In five of the HCs, both the CALIPR and tensor‐valued diffusion acquisitions were repeated after repositioning to assess scan‐rescan repeatability.

For lesion visualization in the MS participants, T_2_‐FLAIR (TR/TE/TI = 8000/310/2400 ms, TSE factor = 110, FOV = 256 × 181 × 256 mm^3^ [AP × RL × FH], acquired resolution = 1 × 1 × 1 mm^3^, reconstructed to 0.67 × 0.67 × 0.67 mm^3^, CS‐SENSE factor = 9, time = 5 min) images were also acquired.

### 2.2 Data processing

MWF maps were generated from the CALIPR data using 3D spatial correlation‐based analysis with two spatial iterations.^15^ Tensor‐valued diffusion data was denoised with MRtrix3's^28^ dwidenoise^29^ and Gibbs ringing removal was performed with mrdegibbs.^30^ Susceptibility correction was done on the tensor‐valued diffusion data using FSL's topup,^31^ with Synb0‐DisCo^32^ used to create undistorted *b* = 0 images where reverse phase‐encoded images were not available. The MS examples presented here did not use Synb0‐DisCo. ElastiX^33^ was used for motion and eddy current correction by extrapolating reference volumes through an open‐source MATLAB library wrapper.^34^, ^35^ Maps of μFA and C_MD_ were generated using QTI+,^36^ a framework based on the existing QTI framework with positivity constraints, using the SDPdc method in MATLAB.^37^ This process also generated traditional DTI metrics using the LTE portion of the tensor‐valued diffusion data alone. Brains were extracted using Advanced Normalization Tools' (ANTs)^38^ antsBrainExtraction tool with the NKI template as a guide.

### 2.3 Atlas creation

Study‐specific metric atlases were created. All 25 HCs' T_1_‐weighted images were formed into a template with ANTs' antsMultivariateTemplateConstruction tool^38^ at a resolution of 1 × 1 × 1 mm^3^. Metric maps of MWF, FA, μFA, and C_MD_ from the HCs were registered to this template and a mean map and a standard deviation map were created for each metric. When considering these atlases, regions of high coefficient of variation (CoV = standard deviation/mean), particularly in the ventricles and around the brain edges, were neglected to soften the impact of interpolation and averaging effects.

To distinguish natural biological variation between participants from measurement noise, scan‐rescan repeatability of MWF, FA, μFA, and C_MD_ was assessed in five healthy participants. WM masks were generated from each participant's T_1_‐weighted images using ANTs Atropos^39^ and warped to each run of MWF, FA, μFA, and C_MD_, and the mean and standard deviation of each measure in WM were calculated. The WM scan‐rescan coefficient of variation was calculated as:

$$\begin{array}{cc} & \text{Scan}-\text{rescan}\;\mathrm{CoV}\\ & \;=\frac{\text{Standard deviation between scan and rescan}}{\text{Mean of scan and rescan}}\\ & \;\times 1.125\times 100\% \end{array}$$

The additional factor of 1.125 accounts for the small number of scans used in the CoV calculation, which can bias the CoV toward smaller values.^40^, ^41^

### 2.4 Correlation analysis

Ten WM structures were identified from the Johns Hopkins University WM tract atlas^42^ and warped into each HC's metric space using ANTs symmetric diffeomorphic normalization (SyN) registration,^38^ and WM masks for each subject were generated using ANTs Atropos with KMeans segmentation.^39^ Using a generic atlas was expected to enable comparisons with prior studies that used similar methods of identifying regions of interest (ROIs) and extracting mean values of measures, despite the fact that it relies heavily on the registration of the atlas to the individual subjects' data. The structures investigated were: genu, body and splenium of the corpus callosum (CC), anterior thalamic radiation (ATR), cingulum (CG), corticospinal tract (CST), major and minor forceps, superior longitudinal fasciculus (SLF), and inferior longitudinal fasciculus (ILF). The mean and SD of each metric was calculated for each structure. Population‐averaged and non‐population‐averaged MWF from all regions were then compared to FA, μFA, and C_MD_ in healthy participants with Spearman correlations, as not all the data was necessarily normally distributed. A Bonferroni correction was used in cases with multiple comparisons.

### 2.5 Tract profiling

To ensure that the same tracts were studied in every subject, a tractography atlas was generated for the subsequent tract‐based analyses using the LTE acquisition from a subset (12/25) of HCs' tensor‐valued diffusion scans. The subset of 12 was randomly chosen for tract atlas construction, while the remaining 13 healthy datasets that were not involved in atlas creation were used to assess tract warping. Each LTE acquisition was upsampled to 1.25 mm^3^ isotropic to improve the fiber orientation distribution (FOD) generation and subsequent tractography, FODs were generated for each subject using a multi‐shell multi‐tissue constrained spherical deconvolution algorithm, and an FOD population template was created using MRtrix3.^28^ Peaks were extracted from the FOD template and used to generate fiber bundles using TractSeg.^43^, ^44^ Tracts were then warped to each individual subject's lower‐resolution tensor‐valued diffusion space with MRtrix3. Tract warping was evaluated manually, by first checking the registration between each subject and the template, and then overlaying the chosen tracts onto the subject's lower‐resolution *b* = 0 image to ensure correct locations.

MWF maps were registered to the 3 mm tensor‐valued diffusion space (by registering a long‐T_2_ echo to the *b* = 0 image) so that all metric maps could be profiled along tracts at the same resolution and location, and all metric maps were masked to remove CSF. Metrics were then profiled using TractSeg's Tractometry tool.^45^, ^46^ As the different metrics had different dynamic ranges, minimum‐maximum (min‐max) normalization was used to visualize trends along the tracts. This normalization was based on the 3 mm HC data and the same minimum and maximum were used for all participants:

$$\begin{array}{cc} & \text{Normalized metri}c_{\text{position}x}\\ & \;=\frac{\text{Subject metri}c_{\text{position}x}-\min\left(\mathrm{HC}\;\text{mean}\;{\text{metric}}_{\mathrm{all}\;\text{positions}}\right)}{\max\left(\mathrm{HC}\;\text{mean}\;{\text{metric}}_{\mathrm{all}\;\text{positions}}\right)-\min\left(\mathrm{HC}\;\text{mean}\;{\text{metric}}_{\mathrm{all}\;\text{positions}}\right)} \end{array}$$

To quantify relationships between metrics through tract profiling, the relationship between each individual metric along the 100 vertices of the tract profile was examined in HCs (specifically for the genu and ATR, for demonstration purposes) and quantified using Spearman's correlations as the relationships were not necessarily one‐to‐one. Principal component analysis (PCA) was performed for these two tracts, using all 25 HCs' 100 tract profile points (resulting in a matrix of 2500 points × 4 measures), and explained variance ratios were calculated for each tract to determine how many PCA components could effectively explain the data variation.

### 2.6 Z‐score maps in MS

The MS metric maps were also warped to the atlas, and a z‐score map was calculated for each measure for each MS participant using:

$$\text{Metric}\;z-\text{score}=\frac{\mathrm{MS}\;\text{metric value}-\mathrm{HC}\;\text{sample mean}}{\mathrm{HC}\;\text{sample standard deviation}}$$

From CoV maps of each of these metrics, regions with CoVs higher than 0.75 were masked out to remove regions of mis‐registration or high natural biological variation.^47^ Z‐score maps were multiplied by masks of low CoV areas and thresholded to include only highly abnormal values (z < −0.5 for MWF, z < −1.96 for μFA/FA, z > 1.96 for C_MD_) to highlight areas of maximum deviation from HCs.

### 2.7 Tract profiling in MS

Tract profiles for the MS participants were generated as described in section 2.4 and were plotted using the same min‐max normalization based on the healthy average as in section 2.4, to best enable comparisons to the healthy average.

## 3 RESULTS

### 3.1 Metric atlases

**FIGURE 1:**
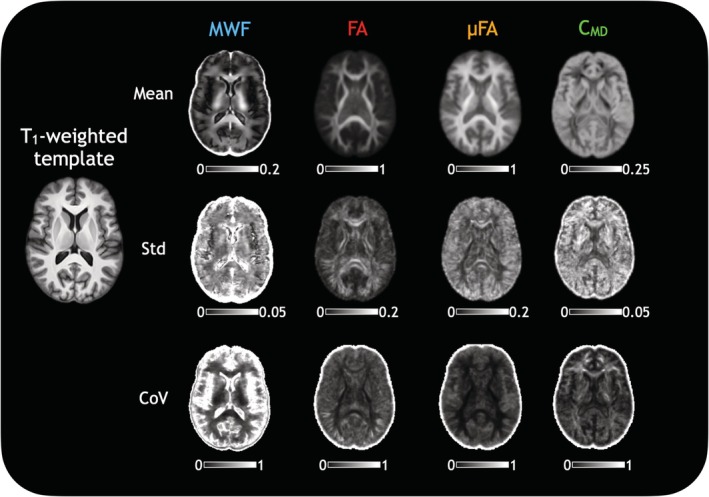
Mean, standard deviation, and coefficient of variation (CoV) atlases of each metric created by averaging together data from 25 healthy participants (11 male/14 female; mean age = 46 years; range = 23–70 years).

**FIGURE 2:**
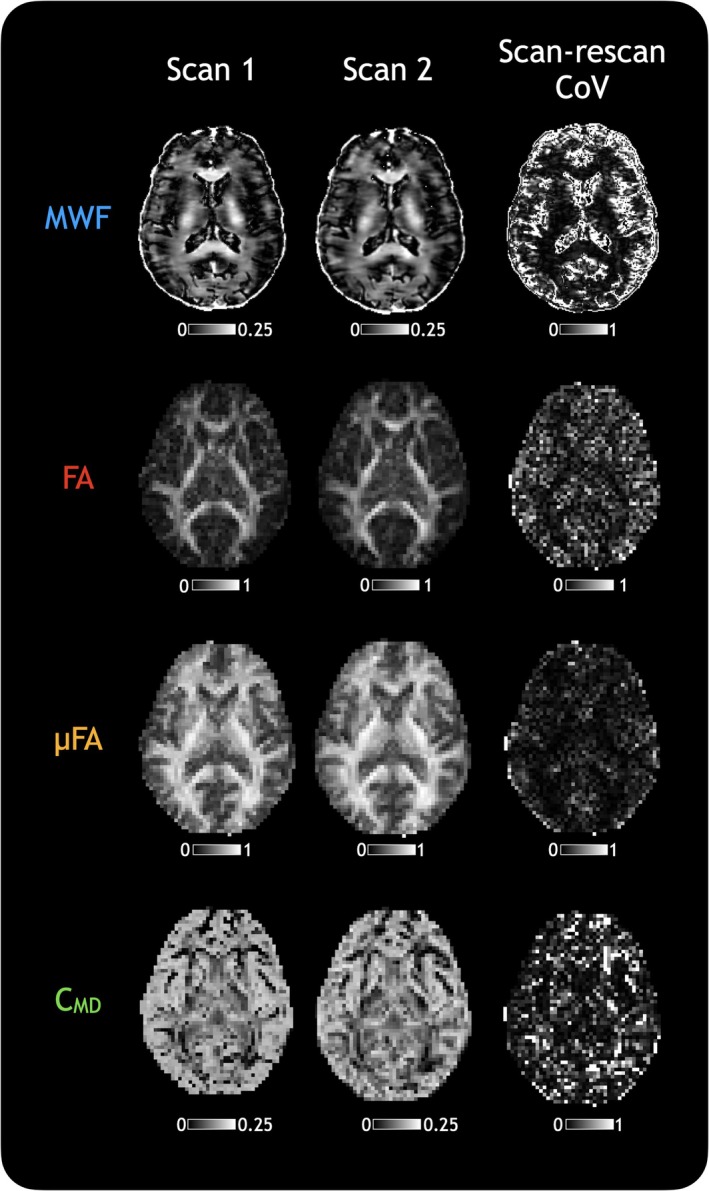
Scan, re‐scan, and the scan‐rescan coefficient of variation (CoV) map in one healthy subject for each of the measures. When considering only white matter, across all five subjects who were scanned twice, the CoV was 1.98% for myelin water fraction (MWF), 1.41% for fractional anisotropy (FA), 2.30% for microscopic FA (μFA), 4.96% for tissue heterogeneity (C_MD_).

Atlases of MWF, FA, μFA, and C_MD_ were created to assess how these metrics behaved on average, and how they varied across the healthy cohort. Figure 1 shows atlases of mean, standard deviation, and CoVs from the HCs for each of these metrics. The mean MWF shows increased myelin content in regions of the T_1_‐weighted image corresponding to WM, as expected. The FA shows increased anisotropy only in tracts of WM that demonstrate low tissue heterogeneity on the C_MD_ map. The μFA shows a more uniform appearance in all regions of WM, although with slightly reduced anisotropy anteriorly, which visually coincides with slightly lower MWF in those same areas. MWF shows the highest variation between subjects (CoV = 43% in WM, with higher CoV in gray matter because of much lower MWF), followed by C_MD_ (CoV = 35%), FA (CoV = 21%), and μFA (CoV = 11%). Scan‐rescan repeatability assessment in WM in five HCs (1 HC shown in Figure 2) showed that all measures were reproducible (average MWF scan‐rescan CoV = 1.98%, FA scan‐rescan CoV = 1.41%, μFA scan‐rescan CoV = 2.30%, C_MD_ scan‐rescan CoV = 4.96%). Given that measures themselves are reasonably repeatable, the spread of metric values in the atlases is likely driven by natural biological variation. The atlases suggest that although myelin content varies between people, tissue anisotropy is a more constant feature of brain architecture across people. Figure S1 shows metric values in all the structures considered in HCs.

### 3.2 Correlation analysis

**FIGURE 3:**
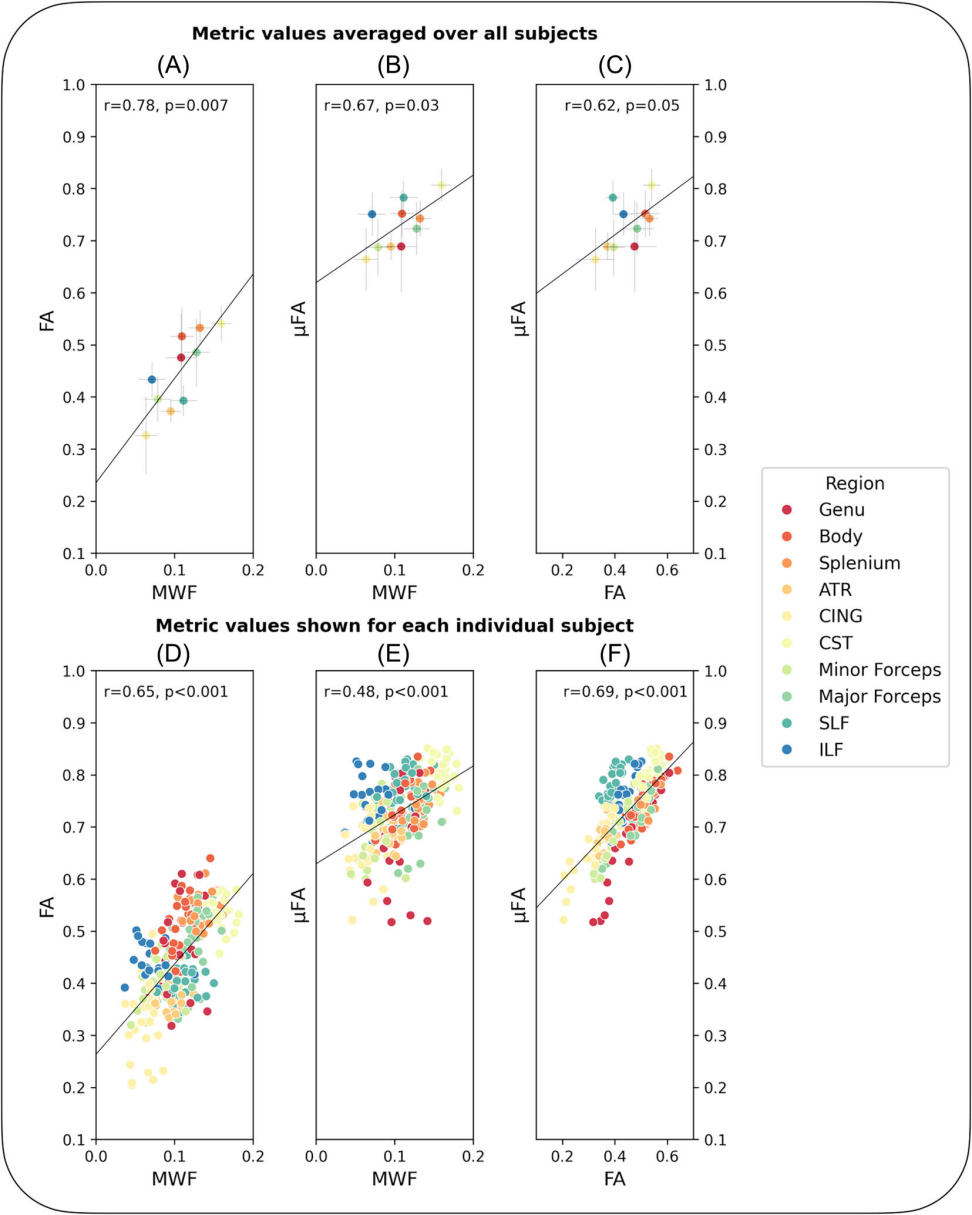
Comparison of myelin water fraction (MWF) and measures of anisotropy at the level of brain structures. (A–C) are averaged over all subjects for each structure and each point corresponds to one structure, with error bars representing the standard deviation of metric values across all subjects. MWF was correlated with fractional anisotropy (FA) (A, Spearman's *r* = 0.78, *p* = 0.007), and less strongly with microscopic FA (μFA) (B, Spearman's *r* = 0.67, *p* = 0.03). (D–F) Show each subject's region of interest values, thereby including the effect of biological variation between subjects. Each point corresponds to one subject's data.

**FIGURE 4:**
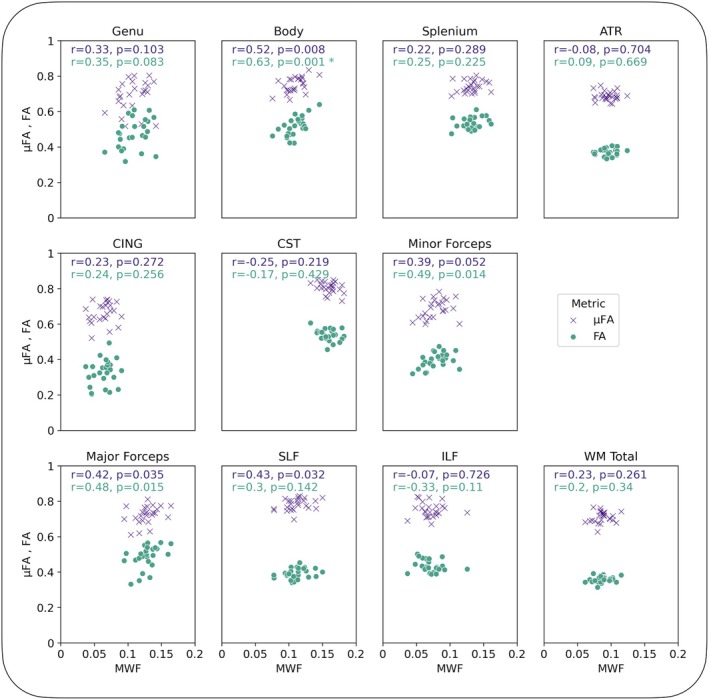
Comparison of myelin water fraction (MWF) and anisotropy measures separated by structure, with each point representing a subject's metric value for that structure. Structures considered were the genu, body and splenium of the corpus callosum (CC), anterior thalamic radiation (ATR), cingulum (CING), corticospinal tract (CST), minor and major forceps, superior longitudinal fasciculus (SLF), and inferior longitudinal fasciculus (ILF). Most regions do not present significant relationships between either microscopic fractional anisotropy (μFA) or fractional anisotropy (FA) with MWF, although structures with higher MWF tend to show higher anisotropy. The multiple comparisons were corrected using a Bonferroni correction (22 comparisons resulting in a significance level of *p* < 0.002, and only one relationship (MWF vs. FA in the body of the CC) met this threshold). * indicates significant relationships (*p* < 0.002).

The relationship between MWF and both measures of anisotropy is presented in Figure 3A–C, with data from each of the 10 structures averaged across all HCs to remove the effect of biological variation. Over all structures, MWF and FA were correlated (*r* = 0.78, *p* = 0.007), whereas the relationship between MWF and μFA was weaker (*r* = 0.67, *p* = 0.03), as was the relationship between FA and μFA (*r* = 0.62, *p* = 0.05). When including data from each subject to account for natural variation between people (Figure 3D–F), some relationships were strengthened, indicating that although the spread in metric values because of biological variation strengthens the correlation, the measures themselves are not all as strongly related. This is further corroborated by Figure 4, where the relationships between MWF and FA and μFA, when separated by structure, show few significant relationships. The relationships with C_MD_ (representing tissue heterogeneity) are presented in Figure S2 for completeness, showing no significant relationships with any of MWF, FA, or μFA when averaged across the whole population.

### 3.3 Tract profiling

**FIGURE 5:**
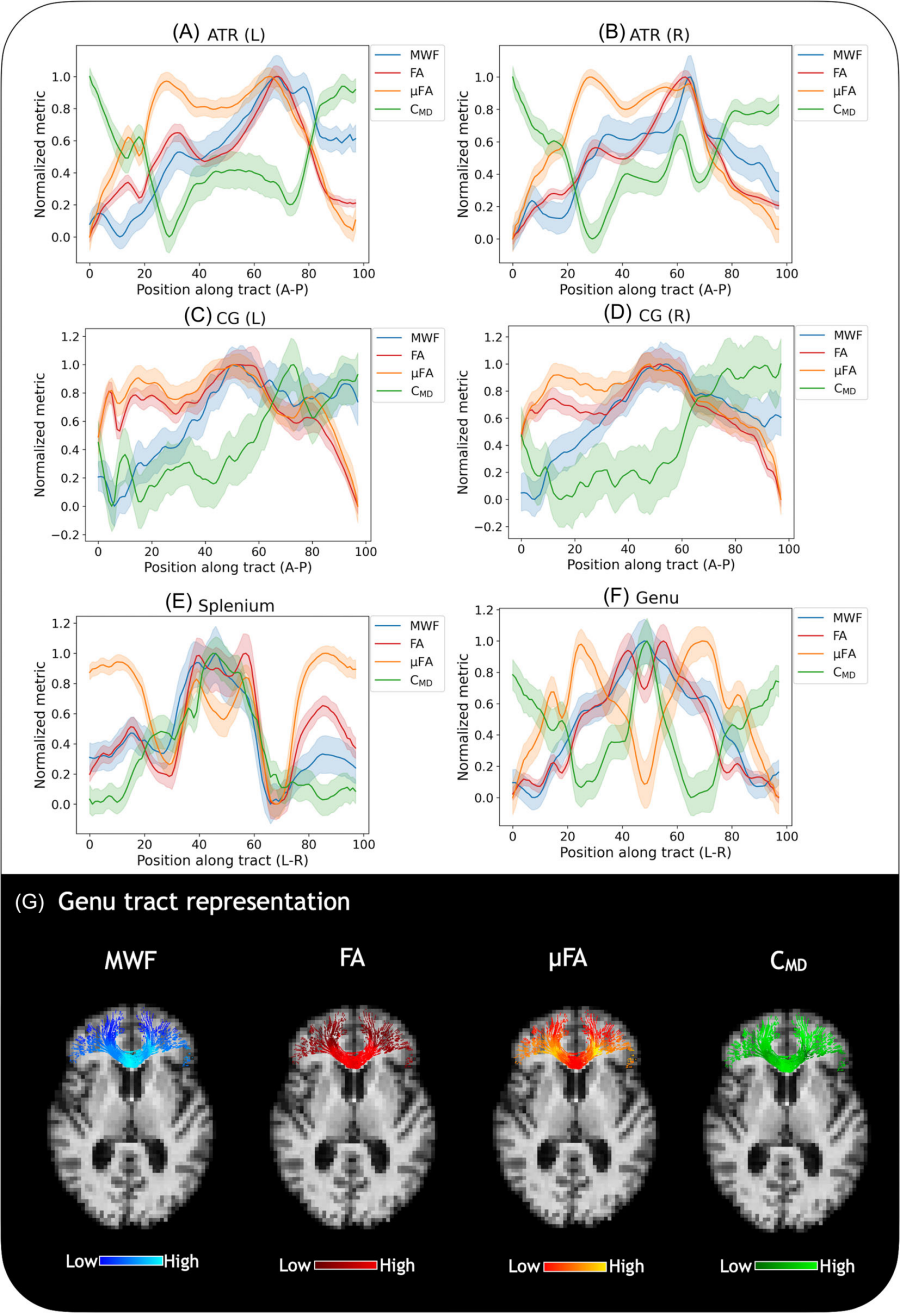
Examples of tract profiling across all healthy participants. All data presented is min‐max normalized to the same mean healthy values, with shaded areas representing a 95% confidence interval. In the anterior thalamic radiation (ATR) and cingulum (CG) (A–D), myelin water fraction (MWF), fractional anisotropy (FA), and microscopic FA (μFA) showed a similar pattern, while tissue heterogeneity (C_MD_) generally demonstrated an opposing relationship. The left and right hemisphere profiles follow similar patterns for each tract. For the splenium and genu (E,F) which cross the midline, there is a noticeable drop in the μFA and a peak in MWF and C_MD_. (G) shows the projection of each measure onto the genu tract overlaid on a healthy participant, with the coloring indicating metric values.

Figure 5 presents min‐max normalized tract profiles for the entire healthy cohort. For the ATR and CG, MWF and anisotropy measures behave similarly, and the left and right hemisphere profiles follow similar patterns for each tract. Some variations along the tract length are visible; for example, the ATR shows distinct peaks and valleys along its length, particularly for μFA and C_MD_. The ATR and CG were chosen to display here as tracts that show left–right symmetry, but did not cross the midline. For the splenium and genu, tracts that cross the midline, there are more distinct drops in the μFA and peaks in MWF and C_MD_ along the tract length. The fairly narrow 95% confidence interval shading suggests that although there may be substantial variation of metrics within structures, these variations are consistent between subjects.

**FIGURE 6:**
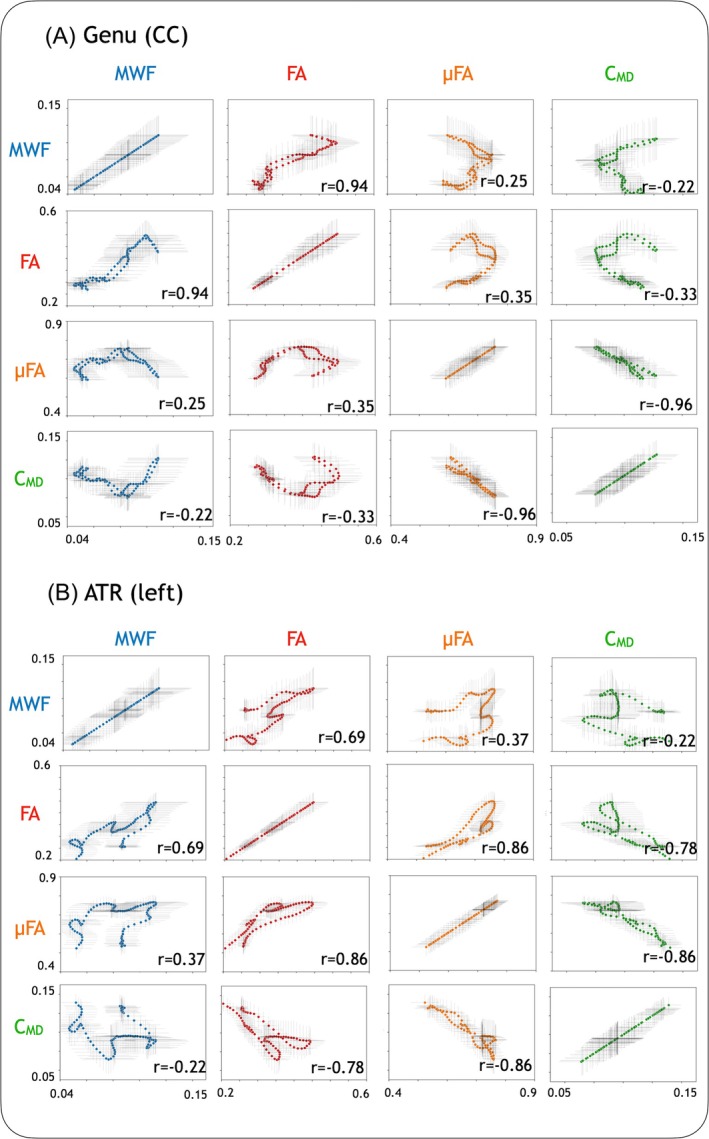
Evaluating metric relationships along the (A) genu and (B) left anterior thalamic radiation (ATR). In each plot, each colored point represents the average of the metric at the same location on the tract from all 25 healthy controls (HCs), with error bars representing the standard deviation in the metric at that point. Spearman's correlations presented on each plot are from the average value of the metrics at each location, and all correlations had *p* < 0.05. Fractional anisotropy (FA) and myelin water fraction (MWF), and microscopic FA (μFA) and tissue heterogeneity (C_MD_), appear strongly related in these tracts in healthy subjects. Along these tracts, the relationships are not necessarily one‐to‐one (e.g., the relationship between MWF and μFA).

Relationships between each individual measure across all 25 HCs have been demonstrated in two tracts in Figure 6, indicating relationships that are not necessarily one‐to‐one. Across both considered tracts, MWF and FA are related (Spearman's *r* = 0.94 in genu, *r* = 0.69 in ATR, *p* < 0.01 for both), and μFA and C_MD_ are related (Spearman's *r* = −0.96 in genu, *r* = −0.86 in ATR, *p* < 0.01). Although the spread of some metric values appears wide, the overall trends and relationships appear to hold even when considering individuals (Figure S3). PCA for both tracts showed that three components are necessary to explain more than 90% of the variance in the data (3 components explained 94% of the variance in genu and 96% of the variance in ATR, see Figure S4), suggesting that three of the four measures provide non‐overlapping information.

### 3.4 Z‐score maps in MS

**FIGURE 7:**
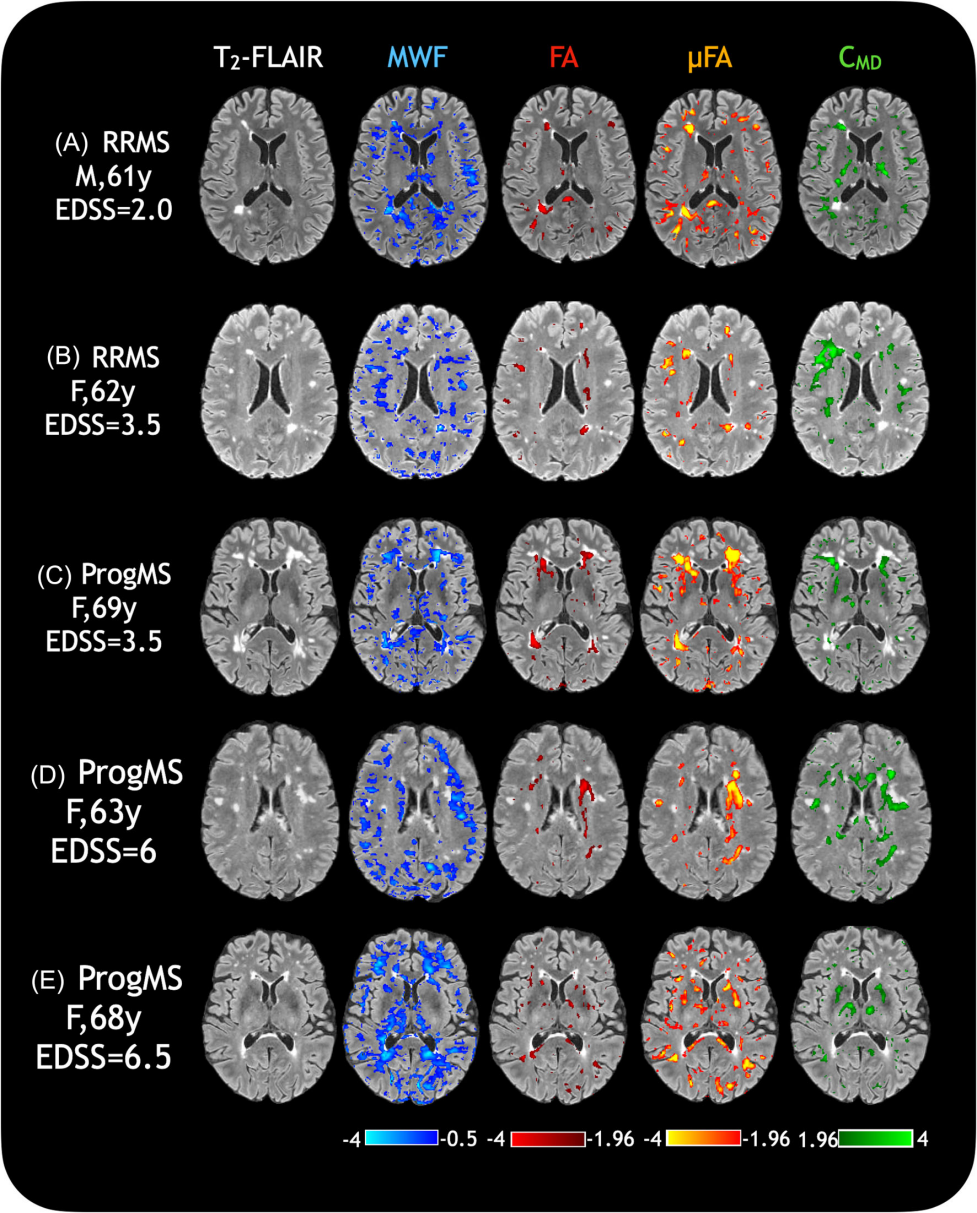
Z‐score maps of the different metrics overlaid on five MS participants' FLAIR images. Lesions show differences in myelin water fraction (MWF), fractional anisotropy (FA), and microscopic FA (μFA) whereas areas surrounding lesions show more differences in MWF, μFA, and tissue heterogeneity (C_MD_).

The five MS participants were compared to the metric atlases using z‐score maps to indicate areas on each metric map that were significantly different from the average healthy population (Figure 7). Note that the MWF z‐score values have a different threshold because of their naturally high variability between healthy individuals. Although MWF and μFA showed decreases in most lesions and normal‐appearing WM (NAWM), these changes were not always the same or to the same extent, indicating differing types or amount of damage. C_MD_ primarily showed increases in peri‐lesional areas and NAWM. FA, although still sensitive to lesions, often reflected characteristics of both MWF and μFA changes, and fewer changes in NAWM. Z‐score histograms without masking out any regions of high variability are presented in Figure S5, for a more even comparison between measures and across subjects.

### 3.5 Tract profiling in MS

**FIGURE 8:**
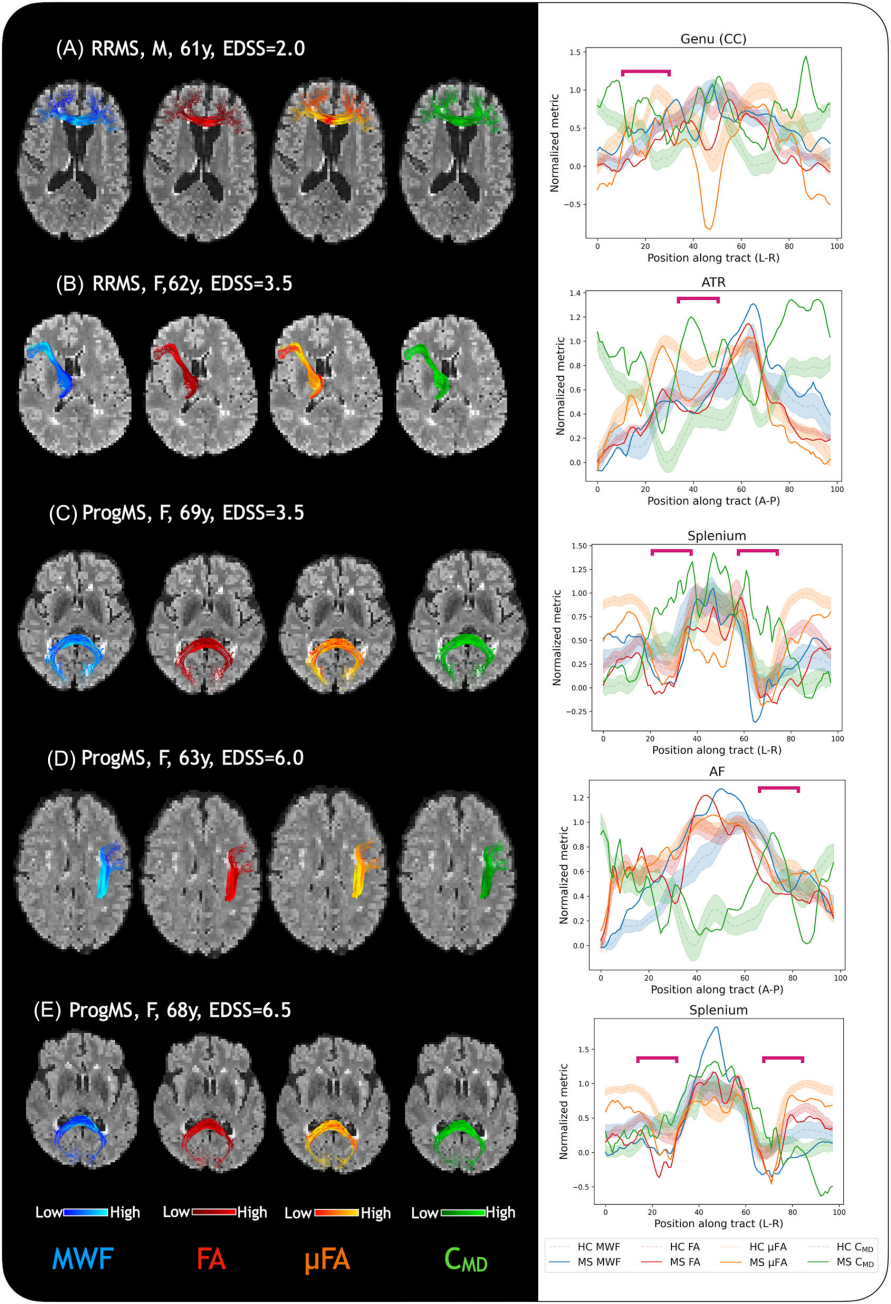
Tract profiles of myelin water fraction (MWF), fractional anisotropy (FA), and microscopic FA (μFA), and tissue heterogeneity (C_MD_) visualized along tracts containing lesions in five participants with multiple sclerosis (MS). The profiles of each measure (colored by measure, with color intensity corresponding to metric value) are overlaid on FLAIR images registered to diffusion space to show their locations relative to lesions. Tract profiles in graphical form are presented on the right, with individual subjects shown as solid lines, while profiles of the healthy average are shown as shaded lines. Regions through and around MS lesions (pink brackets on graphical tract profiles) generally demonstrate lower (μFA, FA, and MWF) and higher (C_MD_) values compared to the healthy average at the same location, and different lesions show different patterns of metric changes.

Tract profiling can offer a clearer understanding of how tracts of WM up‐ and down‐stream from a disruption such as lesion may be affected. By assessing metrics along WM tracts connected to a lesion, different types of damage, as well as impending changes to other regions along the same tract may become evident. Figure 8 shows examples of the tract profiling technique for the five MS participants along lesional tracts (lesions are marked by pink brackets). Across the MS examples (Figure 8A–E), there is considerable variability in how the measures differ, with noticeable changes to MWF (Figure 8D), μFA (Figure 8A,B), or both (Figure 8C,E), which may be an indicator of differenttypes of damage. C_MD_ appears to be a sensitive marker of damage along the entire tract, showing higher tissue heterogeneity along most lesional tracts compared to the healthy average. FA again appears to mirror both MWF and μFA in different parts, which may be useful if either MWF or μFA were not acquired.

## 4 DISCUSSION

Our preliminary investigation comparing MWF and measures from tensor‐valued diffusion imaging reveals substantial and consistent tract‐specific pattern differences between metrics. In healthy tissue, MWF and μFA oftenshow similar patterns, but inconsistencies arise in regions such as the CC, with FA reflecting aspects of both measures, and C_MD_ often behaving inversely to μFA. We then investigated how these measures and the relationships between them were affected in tissue undergoing different pathological processes in MS and found disruptions in tract patterns and metric relationships.

### 4.1 Correlation analysis

Correlation analysis of metric values averaged across all HC, regions of WM that are highly coherently oriented (i.e., CC, ATR) show high anisotropy (FA, μFA), high myelin content (MWF) and generally low heterogeneity (C_MD_) (Figure 1), in agreement with qualitative comparisons in Lampinen et al.^48^ This was expected in healthy brain, where axonal density and myelin content are linked,^21^, ^49^ and measures of anisotropy were expected to be related to MWF, although not directly driven by myelination. It is known that FA is sensitive to a range of microstructural features such as fiber orientation dispersion, anisotropy, fiber diameter, packing density, and possibly myelin content.^50^, ^51^, ^52^ The relationship between myelin content and anisotropy (as given by MWF and FA) has been previously characterized as weak to strong^23^, ^24^, ^51^, ^53^ when comparing the measures across multiple brain structures, but weak when considering structures separately.^23^, ^53^ Correlation analysis of data from our study is in line with these previous observations (Figures 3 and 4). Such an instance of Simpson's paradox, where a relationship breaks down when looking at sub‐components of the data, may actually point to a slight myelination bias in FA: FA is highest in areas of highly coherently aligned WM, which are also likely to have higher myelin content. Conversely, FA is lower in regions of more disperse fiber orientation, which may also have a variety of axon diameters and myelin thicknesses, resulting in overall lower myelin content.^54^, ^55^, ^56^, ^57^ Therefore, when looking across many different structures with various levels of myelination, this results in a moderate relationship between FA and MWF, which subsequently breaks down when looking at separate structures. A structure‐wise comparison of FA and μFA is presented in Figure S6 for completeness. Anisotropy is thought to be independent of myelination,^52^, ^58^, ^59^ and the relationship between MWF and μFA is generally weaker, which may reflect μFA being a marker of local anisotropy with fewer confounding features. From these correlation analyses, it can be concluded that MWF represents myelin content, μFA represents local anisotropy, and as expected, FA represents anisotropy with other associations such as myelination and confounds such as orientation dispersion.^21^

### 4.2 Tract profiling

Profiling the patterns of MWF, FA, μFA, and C_MD_ along WM tracts was used to better understand the relationship between myelin content, anisotropy, and tissue heterogeneity, as comparing metrics averaged over entire structures does not convey the complexity of tissue microstructure within them. With tract profiling, metrics were extracted from the same spatial positions in all subjects and min‐max normalized. In tracts such as the ATR, CG, and others that did not cross the midline (Figure 5A–D), the left and right hemisphere tract profiles were similar. Tract profiles of μFA, FA, and MWF followed similar patterns, showing that anisotropy may possibly be influenced by myelin content in these tracts, whereas C_MD_ demonstrated the opposite relationship. However, in regions that crossed the midline, such as the splenium and genu of the CC (Figure 5E,F, midline position 40–60), a decrease in μFA was accompanied by a peak in MWF and C_MD_, and a rise and valley in FA. This behavior of MWF and FA is similar to previous observations in callosal regions,^22^ and further points to FA's potential association with myelination. The increase in MWF with decreased μFA, however, was unexpected and may reflect unusual tissue microstructure in the genu and splenium where an increase in fiber orientation dispersion at the midline of the CC was previously observed using Polarized Light Imaging and histology^60^; this feature is corroborated by an increase in C_MD_. C_MD_ may, therefore, add useful information on tissue heterogeneity to help better resolve microstructural features.

While Figures 3 and 4 investigate correlations between MWF, FA, μFA, and C_MD_ on a per subject and a per structure basis, Figures 5 and 6 interrogate much smaller and presumably more internally uniform brain regions providing a finer microstructure probe. Observation of parameter relationships along the genu (CC) and left ATR fiber tracts revealed unexpected results in some cases. Consistent with the Figures 3 and 4 of whole structure‐based measurements, the MWF versus FA plot in Figure 6 followed approximately straight‐line behavior along each tract. The μFA versus C_MD_ trajectories also showed roughly straight‐line behavior, but this could not have been anticipated from the unimpressive per structure and per subject plots of μFA versus C_MD_ in Figure S2B,E. Finally, and perhaps the most intriguing, the MWF versus μFA and FA versus C_MD_ trajectories in Figure 6 both exhibit complicated multi‐valued curves along both the left ATR and genu (CC) tracts. More research is needed before the full value of this tract‐based microstructural probe can be assessed.

### 4.3 Demonstration in MS

The ability of these measures in combination to describe tissue microstructure more completely was evaluated in five example cases of MS. Based on histological studies, it was expected that WM myelin content and axonal density would be correlated, with some breakdown in this relationship in pathology.^61^ From z‐score maps and tract profiling in MS, differences in lesion and NAWM pathologies were reflected by a variety of changes to our MR metrics. Some lesions showed decreases in only MWF, only μFA, or both, whereas C_MD_ appeared to be a sensitive marker of tissue heterogeneity. FA was sensitive to the presence of lesions and some NAWM damage, generally reflecting changes detected by μFA and occasionally also MWF. Tract profiling may show not only lesional damage, but also changes further along the tract such as contralateral NAWM degeneration, as in Werring et al^62^ and Simon et al,^63^ with the potential to identify locations of early damage not yet detectable with conventional MRI or regions of high vulnerability. In Figure 8A in particular, although the lesion location itself showed reduced μFA and increased C_MD_, further along the same tract also shows similar features without the presence of a lesion, suggestive of downstream damage. In Figure 8C,E, both of which are examples of lesions in both hemispheres along the same tract (splenium), the types of damage in both locations appear to mirror each other, suggesting that perhaps damage in one point along a tract may manifest the same way further downstream along the same tract. Exploring such variability in pathology requires a combination of measures, and if both myelin water and tensor‐valued diffusion data are available, the combination of MWF, μFA, and C_MD_ may present a more complete understanding of pathological changes to tissue. Evaluating more MS cases will allow for more conclusive insights into pathology.

### 4.4 Limitations

There were a number of limitations in our study that warrant consideration. First, the 3 mm isotropic resolution of the diffusion scans made partial volume effects particularly relevant for tract profiling and may have contributed to the discrepant behavior of the metrics in Figure 5E,F. Although CSF was masked out, as the voxels were 27 mm^3^ in size, partial volume effects and leftover contamination from CSF in the genu and splenium (and other parts of the CC) could have led to decreased μFA and increased C_MD_; however, given that FA was derived from the same dataset, it would be expected to be equally affected by partial volume effects, but did not decrease as much at the midline. Tracts extending toward gray matter may also have partial volume effects with gray matter at the gray/white boundary. To rule out partial volume effects, a higher resolution acquisition (2.25 mm isotropic, ˜43% volume of the original voxels) was performed on one subject and showed similar results (Figure S7). This higher resolution acquisition had approximately one‐third of the SNR as the original, potentially biasing the estimates of the metrics. The QTI metrics are known to be affected by low SNR^64^ and provided slightly different values than the lower resolution version, but for purposes of qualitative comparison, this was deemed sufficient. The observed differences between the higher and lower resolution acquisitions are because of the reduced voxel size (accounting for some partial volume effect), but the above‐described tract profile patterns generally persist, pointing to real differences in metric behavior.

The tract atlas‐based approach involved warping tracts from an atlas to each subject to ensure that the same locations were being studied in every subject, and warping was evaluated manually. Although this can affect reproducibility of this approach in other studies and datasets, none of the subjects where tract warping was evaluated needed further corrections (including the 5 MS participants), suggesting that tract warping may be fairly robust.

TractSeg‐derived ROIs based on the diffusion data could also have been used to add specificity to the structures used in the correlation analyses, rather than using ROIs from the JHU‐ICBM atlas. However, the drawbacks related to ROI analysis, such as losing fine detail by averaging over whole structures, still remain. As tractometry can show more localized behavior of measures, it may be more informative, particularly in pathology, as evidenced by the MS tract profiles that show changes to measures along lesional tracts.

Frameworks such as the standard model^65^, ^66^ or diffusion kurtosis imaging^67^, ^68^ also aim to disentangle the signal contributions that tensor‐valued diffusion aims to resolve. The standard model provides an overarching framework for various signal models and representations, with tensor‐valued diffusion being a special case of the framework. Although in this study we compared metrics from only diffusion tensor and tensor‐valued diffusion imaging, future analyses could include applying these other frameworks to the same data.

Although demonstrating MWF, FA, μFA, and C_MD_ in five people with MS was meant to show some of the possible changes to tissue, the limited number of participants still means that many types of changes may not have been visible, and more participants are needed to draw more meaningful conclusions.

Myelin content is known to be related to age. Previous studies have shown that the MWF in most brain structures between age 30 to 70 (forming most of the test population in this study) remains relatively flat,^69^, ^70^ and differences seen because of natural biological variation between subjects were in fact found to be larger than those related to age.^70^ Our healthy cohort had an even spread of ages over a large range (25 subjects; mean age 46 years; SD 15 years; range 23–70 years), so it was expected that any bias because of age variation would be minimal on the relationships explored in this study, and the presence of the same metric trends across all subjects confirms this. Although the MS participants were older than the mean age of the healthy cohort, differences in metrics between the mean age (46 years) and participant ages are smaller than the deviations seen through z‐score maps for these MS participants.^69^, ^70^ The MWF may also be affected by exchange,^6^, ^71^ and regions with faster exchange may result in lowered MWF values, which could impact the relationships found in this study. Other MWF confounds include the presence of myelin debris^5^, ^72^ and iron concentration.^73^

## 5 CONCLUSION

Our study used myelin water and tensor‐valued diffusion imaging to characterize healthy brain tissue. Tract profiling analysis provided a more comprehensive view of tissue microstructure compared to correlation analysis. Metric atlases suggest that anisotropy may be a more fundamental, consistent characteristic of brain architecture common to all healthy individuals, whereas myelination levels vary substantially between people. In five example cases of MS, both tract profiling and z‐score map analysis revealed different patterns of metric changes suggestive of varying types of tissue damage. Given that each measure showed different patterns for different regions and tissue structures, no single metric would be adequate to fully explore microstructural changes occurring within the CNS. Using MWF, μFA, and C_MD_ to understand myelination, anisotropy, and tissue heterogeneity separately with myelin water and tensor‐valued diffusion imaging will be important for further investigating neurological development, aging, disease, and injury.

## FUNDING INFORMATION

Natural Sciences and Engineering Research Council (NSERC) Canada Graduate Scholarship‐ Doctoral (to S.B.); NSERC, Grants/Award Numbers: RGPIN‐2018‐23904 (to S.H.K.) and RGPIN‐2015‐04513 (to A.L.M.), MS Canada Catalyst Research, Grant/Award Number: 920406; Canadian Institutes of Health Research, Grant/Award Number: 178273; Michael Smith Health Research BC (to S.H.K)

## CONFLICT OF INTEREST STATEMENT

None of the authors have a conflict of interest with this work. E.L.M. and G.G. have been employed by Philips Healthcare Canada as MR Clinical Scientists.

## Supporting information


**Figure S1.** Mean and standard deviation values of each metric for the genu, body and splenium of the corpus callosum (CC), anterior thalamic radiation (ATR), cingulum (CING), corticospinal tract (CST), minor and major forceps, superior longitudinal fasciculus (SLF), and inferior longitudinal fasciculus (ILF), presented as mean (bigger font) and standard deviation (smaller font).
**Figure S2.** Comparison of tissue heterogeneity (C_MD_) with myelin water fraction (MWF), microscopic FA (μFA), and fractional anisotropy (FA) at the level of a region of interest. (A–C) are averaged over all subjects for each region of interest (ROI) and each point corresponds to one ROI, with error bars representing the standard deviation of metric values across all subjects. C_MD_ was not significantly correlated with any other measure. (D–F) show each subject's ROI values, thereby including the effect of biological variation between subjects. Each point corresponds to one subject.
**Figure S3.** Individual relationships along the genu visualized as in Figure 6 for one single individual, showing that the same trends in metrics along a tract follow even on a one‐subject basis (e.g., the fairly linear relationship between myelin water fraction [MWF] and fractional anisotropy [FA], and the non‐one‐to‐one relationship between MWF and microscopic FA [μFA]).
**Figure S4.** For two tracts (genu and left anterior thalamic radiation [ATR]), principal component analysis (PCA) was used to derive explained variance ratios to determine how many components would be necessary to explain the dataset of 4 measures. In (A), it was found that for both tracts, 3 principal components were adequate to explain the data when considering healthy controls (HCs), although 2 components were often enough to explain over 85% of the data variance. When assessing Spearman's correlations for both tracts across all 25 HCs in (B), a strong relationship was found between microscopic FA (μFA) and tissue heterogeneity (C_MD_). However in (C), when considering only data from the 5 multiple sclerosis (MS) participants along these two tracts, the explained variance ratios, while still supporting that 3 out of 4 components were adequate to explain the data, showed that the third component became relatively more important. Spearman's correlations in (D) show that the relationship between μFA and C_MD_ becomes weaker in MS, suggesting that these measures may both be useful in pathology.
**Figure S5.** Z‐score maps for each multiple sclerosis (MS) participant, for each measure, presented in the form of histograms. The x‐axis in each plot represents z‐scores (showing z < 0 for myelin water fraction [MWF], fractional anisotropy [FA] and microscopic FA [μFA], and *z* > 0 for tissue heterogeneity [C_MD_]). In these histograms, no additional masking other than CSF masking was performed, thereby allowing the most even comparison between z‐scores of different metrics. The vertical line in each plot indicates where the z‐score maps were thresholded in the main paper (Figure 7).
**Figure S6.** Comparison of fractional anisotropy (FA) and microscopic FA (μFA) separated by structure, with each point representing a subject's metric value for that structure. The multiple comparisons were corrected using a Bonferroni correction (resulting in a significance level of *p* < 0.004, and all relationships met this threshold). (*) indicates *p* < 0.004.
**Figure S7.** Original (3 mm, lower resolution LRES, dashed line) and higher (2.25 mm, Higher Resolution HRES, solid line) resolution image tract profiles in one healthy participant, separated by metric and presented without normalization for comparison. In the splenium, the main valley of microscopic fractional anisotropy (μFA) (C, position 40–60) is less dramatic in the HRES image than LRES although it is still present; tissue heterogeneity (C_MD_) shows a corresponding rise (D), while myelin water fraction (MWF) stays relatively stable through position 40–60 (A, B). In the genu, the valley in μFA is less strong (E–H, position 40–60) in the HRES than the LRES while myelin water fraction (MWF) stays similarly high through those positions. In both the genu and splenium, fractional anisotropy (FA) follows the same patterns at both resolutions. For both tracts, the HRES MWF and LRES MWF were derived from the same MWF dataset resampled to match the resolution of the tensor‐valued diffusion data.

## Data Availability

Processing code is available online at https://github.com/sharadab/microstructure_processing_paper.
