# Supplementary material for: Myelin water and tensor‐valued diffusion imaging: (How) are they related?
Source: Magn Reson Med. 2025 Jul 1;94(5):2038–56. doi: 10.1002/mrm.30620 (PMC12393197; doi:10.1002/mrm.30620)
Supplement: Supplementary file 1 — Figure S1. Mean and standard deviation values of each metric for the genu, body and splenium of the corpus callosum (CC), anterior thalamic radiation (ATR), cingulum (CING), corticospinal tract (CST), minor and major forceps, superior longitudinal fasciculus (SLF), and inferior longitudinal fasciculus (ILF), presented as mean (bigger font) and standard deviation (smaller font). Figure S2. Comparison of tissue heterogeneity (CMD) with myelin water fraction (MWF), microscopic FA (μFA), and fractional anisotropy (FA) at the level of a region of interest. (A–C) are averaged over all subjects for each region of interest (ROI) and each point corresponds to one ROI, with error bars representing the standard deviation of metric values across all subjects. CMD was not significantly correlated with any other measure. (D–F) show each subject's ROI values, thereby including the effect of biological variation between subjects. Each point corresponds to one subject. Figure S3. Individual relationships along the genu visualized as in Figure 6 for one single individual, showing that the same trends in metrics along a tract follow even on a one‐subject basis (e.g., the fairly linear relationship between myelin water fraction [MWF] and fractional anisotropy [FA], and the non‐one‐to‐one relationship between MWF and microscopic FA [μFA]). Figure S4. For two tracts (genu and left anterior thalamic radiation [ATR]), principal component analysis (PCA) was used to derive explained variance ratios to determine how many components would be necessary to explain the dataset of 4 measures. In (A), it was found that for both tracts, 3 principal components were adequate to explain the data when considering healthy controls (HCs), although 2 components were often enough to explain over 85% of the data variance. When assessing Spearman's correlations for both tracts across all 25 HCs in (B), a strong relationship was found between microscopic FA (μFA) and tissue heterogeneity (CMD). However in (C), [file MRM-94-2038-s001.docx]

Supporting Information for

**Myelin water and tensor valued diffusion imaging: (how) are they related?**

Sharada Balaji, Adam V. Dvorak, Neale Wiley, Erin L. MacMillan, Anthony Traboulsee, Irene M. Vavasour, Guillaume Gilbert, G.R. Wayne Moore, David K.B. Li, Cornelia Laule,
Alex L. MacKay, Shannon Kolind


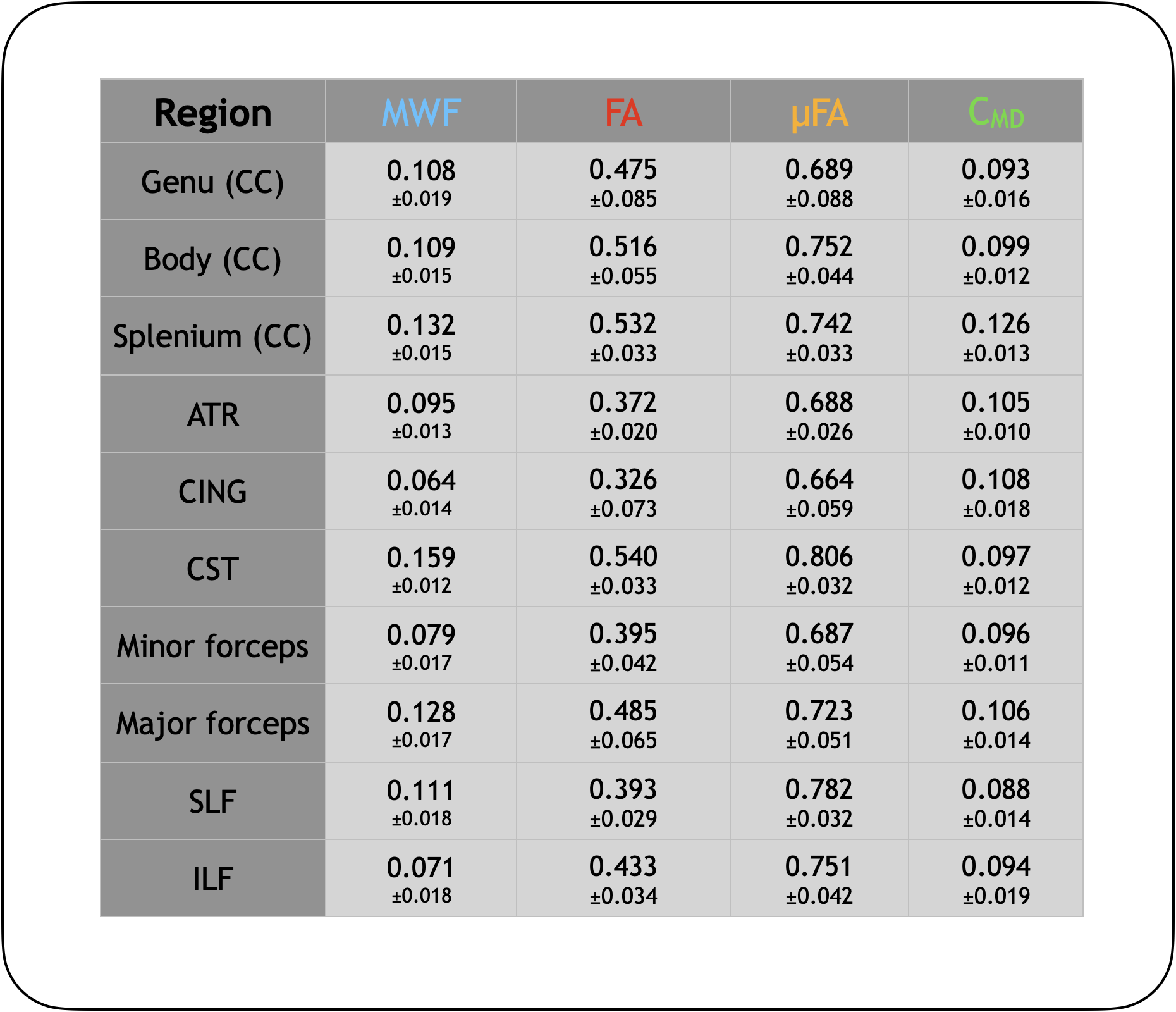


**Figure S1.** Mean and standard deviation values of each metric for the genu, body and splenium of the corpus callosum (CC), anterior thalamic radiation (ATR), cingulum (CING), corticospinal tract (CST), minor and major forceps, superior longitudinal fasciculus (SLF), and inferior longitudinal fasciculus (ILF), presented as mean (bigger font) and standard deviation (smaller font).


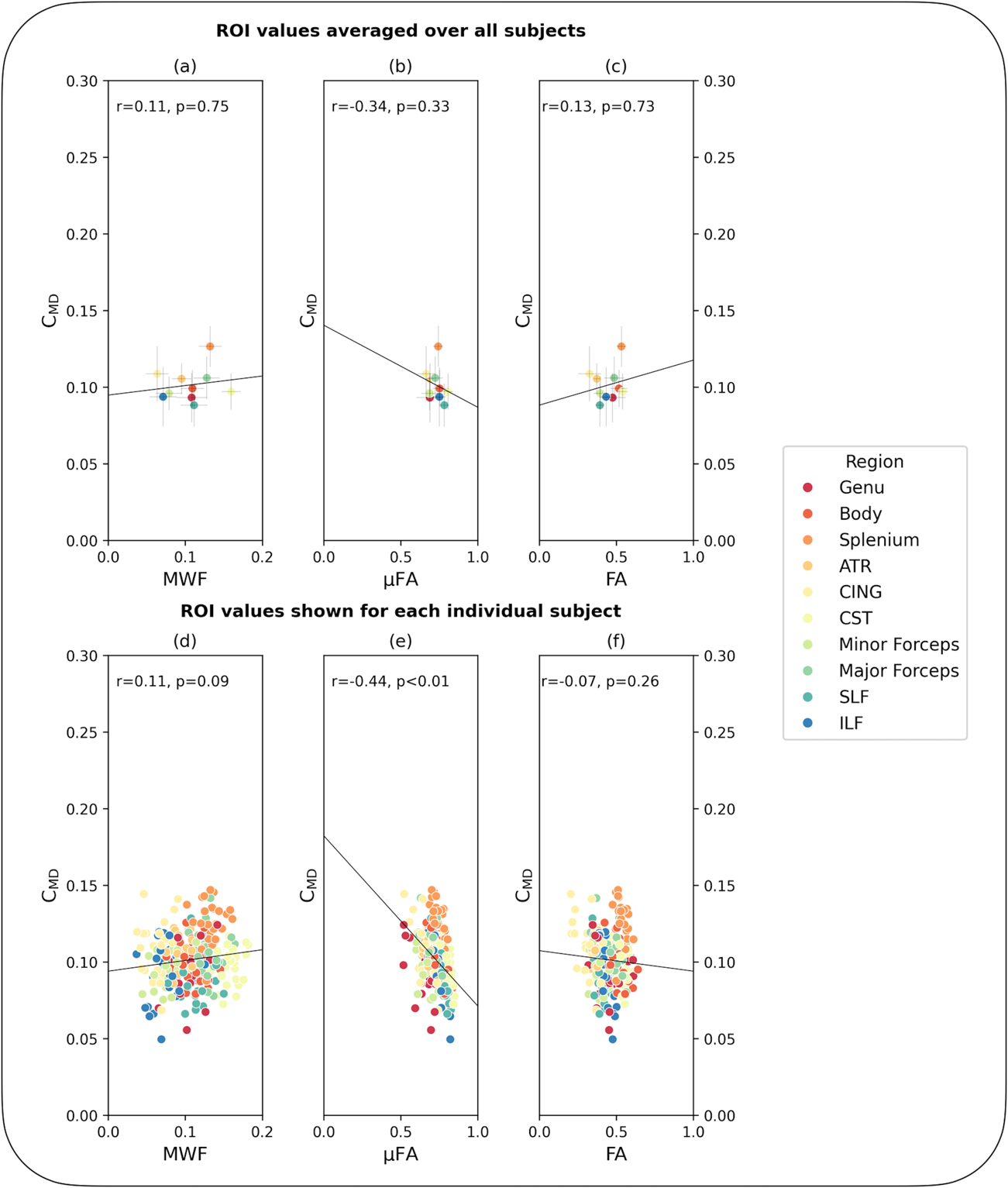


**Figure S2.** Comparison of C_MD_ with MWF, µFA and FA at the level of a region of interest. (a), (b), (c) are averaged over all subjects for each region of interest (ROI) and each point corresponds to one ROI, with error bars representing the standard deviation of metric values across all subjects. C_MD_ was not significantly correlated with any other measure. (d), (e), (f) show each subject’s ROI values, thereby including the effect of biological variation between subjects. Each point corresponds to one subject.


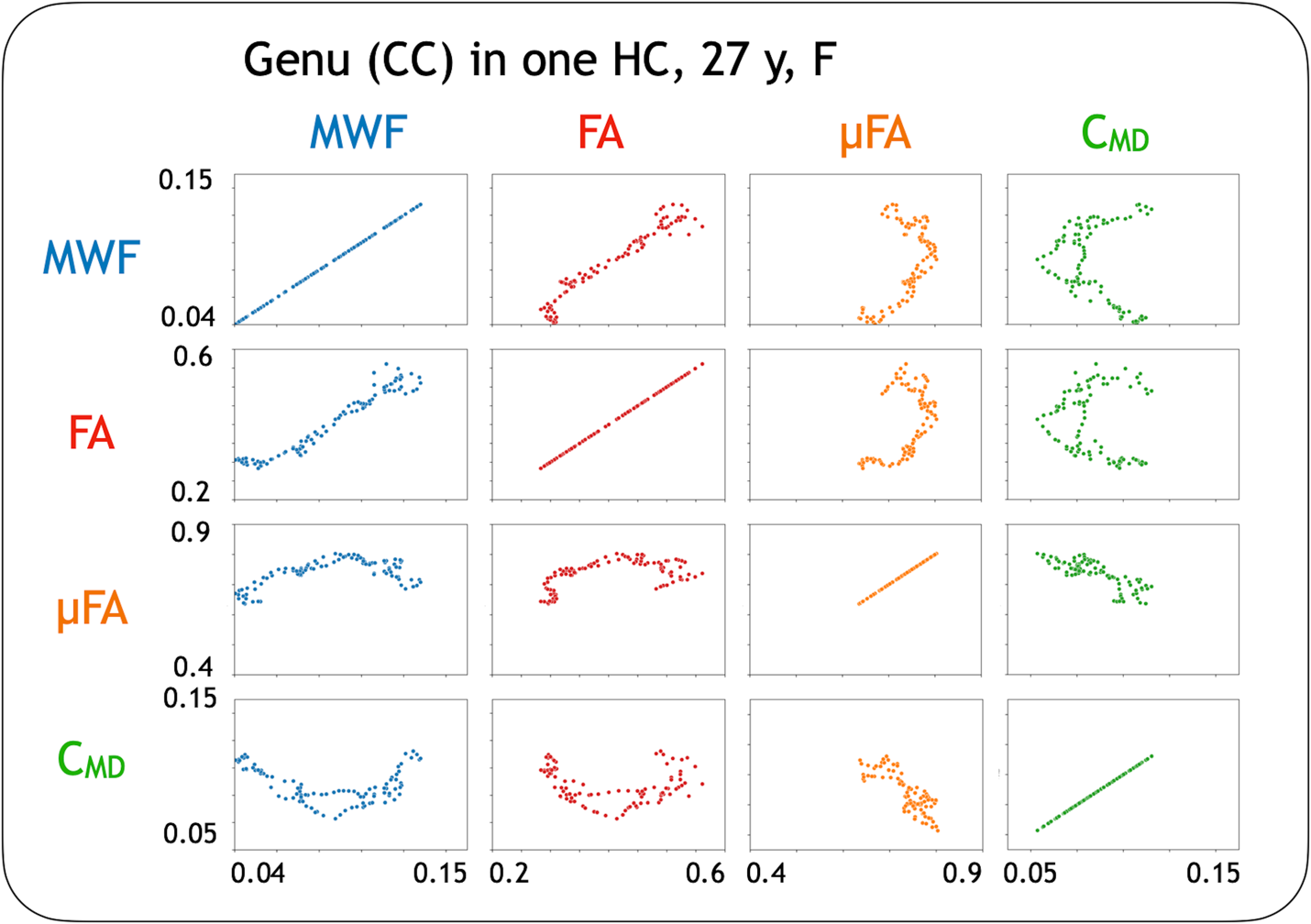


**Figure S3.** Individual relationships along the genu visualized as in Figure 6 for one single individual, showing that the same trends in metrics along a tract follow even on a one-subject basis (e.g. the fairly linear relationship between MWF and FA, and the non-one-to-one relationship between MWF and µFA).


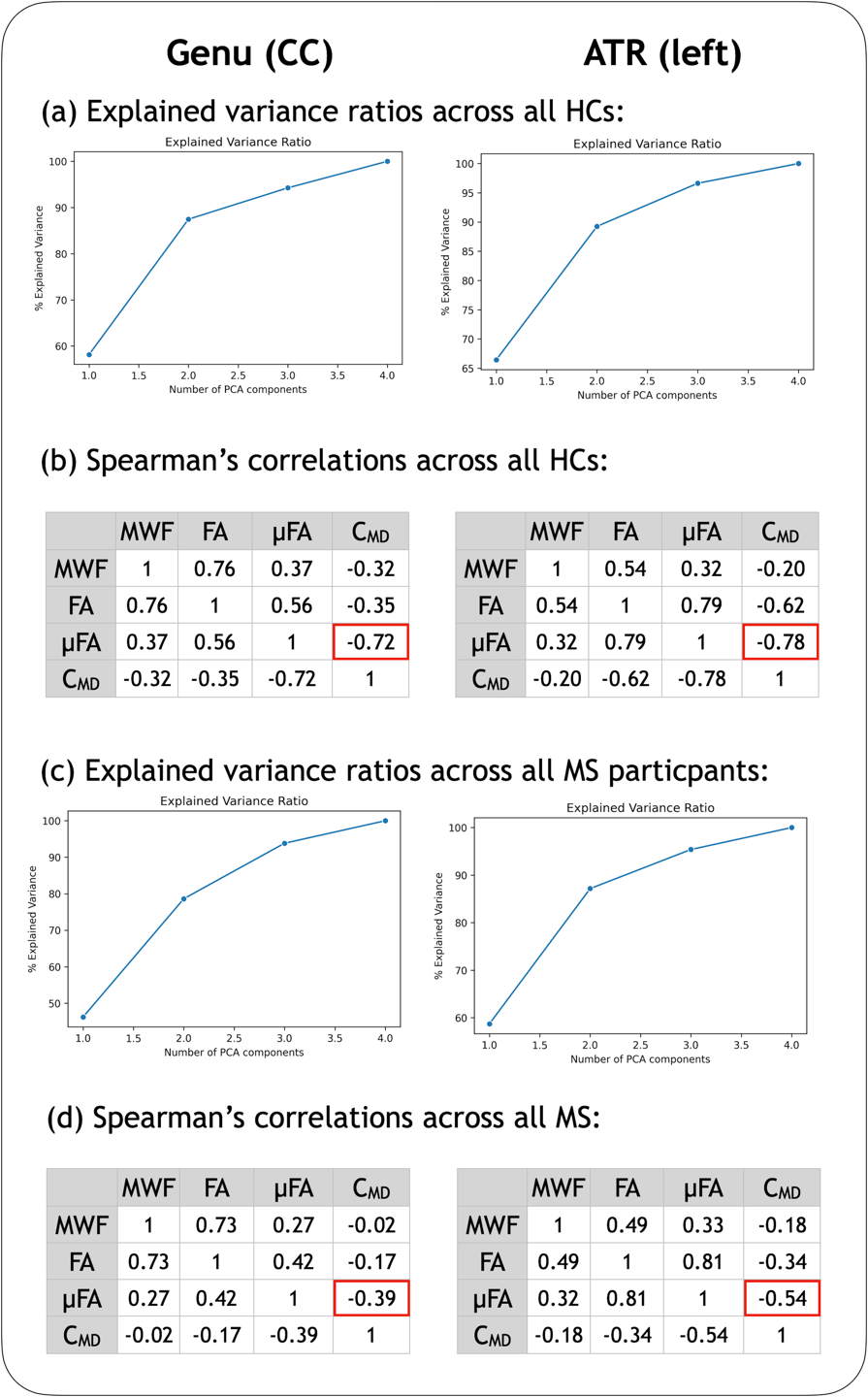


**Figure S4.** For two tracts (genu and left ATR), PCA was used to derive explained variance ratios to determine how many components would be necessary to explain the dataset of 4 measures. In (a), it was found that for both tracts, 3 principal components were adequate to explain the data when considering HCs, although 2 components were often enough to explain over 85% of the data variance. When assessing Spearman’s correlations for both tracts across all 25 HCs in (b), a strong relationship was found between µFA and C_MD_. However in (c), when considering only data from the 5 MS participants along these two tracts, the explained variance ratios, while still supporting that 3 out of 4 components were adequate to explain the data, showed that the third component became relatively more important. Spearman’s correlations in (d) show that the relationship between µFA and C_MD_ becomes weaker in MS, suggesting that these measures may both be useful in pathology.


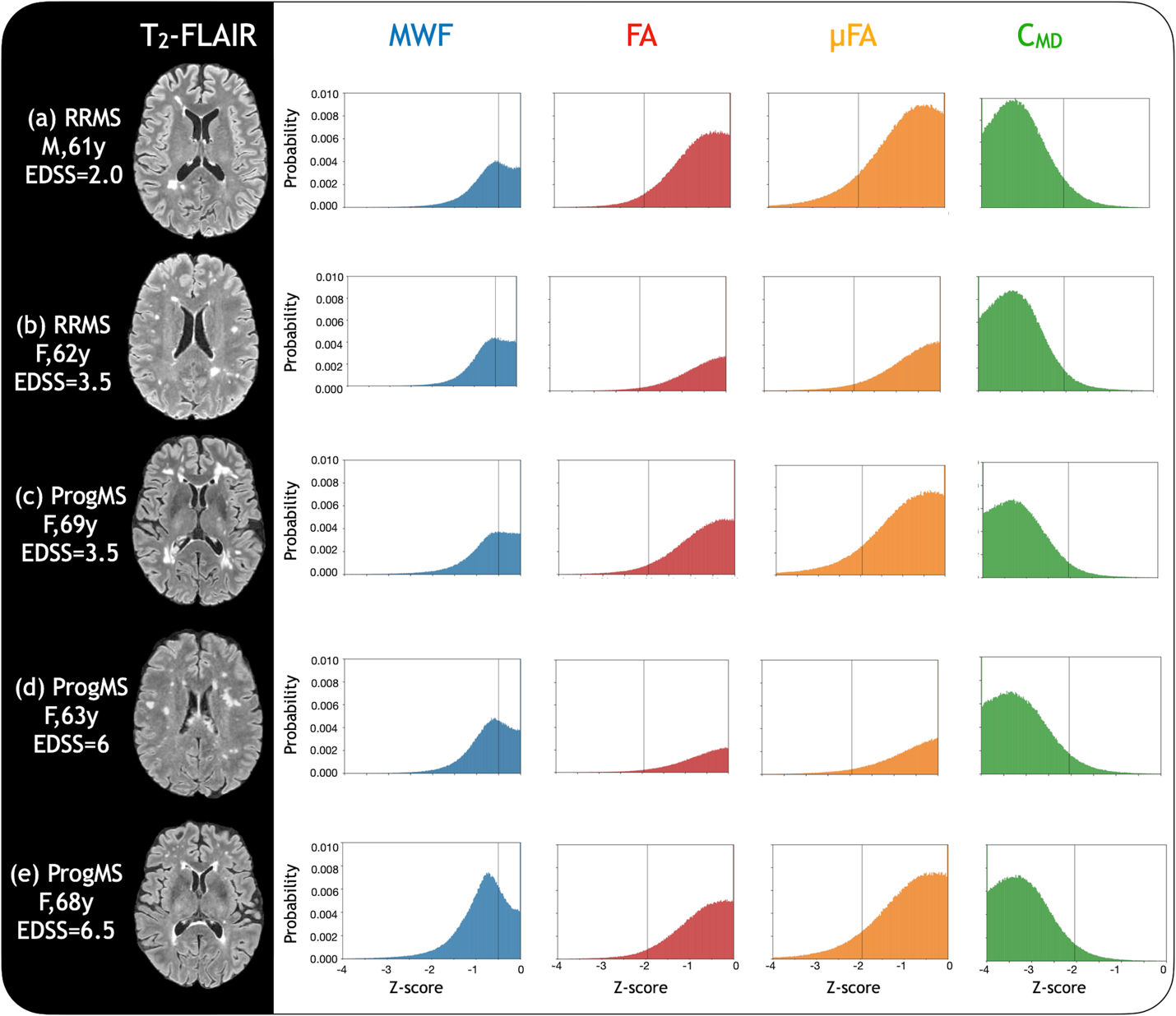


**Figure S5.** Z-score maps for each MS participant, for each measure, presented in the form of histograms. The x-axis in each plot represents z-scores (showing z < 0 for MWF, FA and µFA, and z > 0 for C_MD_). In these histograms, no additional masking other than CSF masking was performed, thereby allowing the most even comparison between z-scores of different metrics. The vertical line in each plot indicates where the z-score maps were thresholded in the main paper (Figure 7).


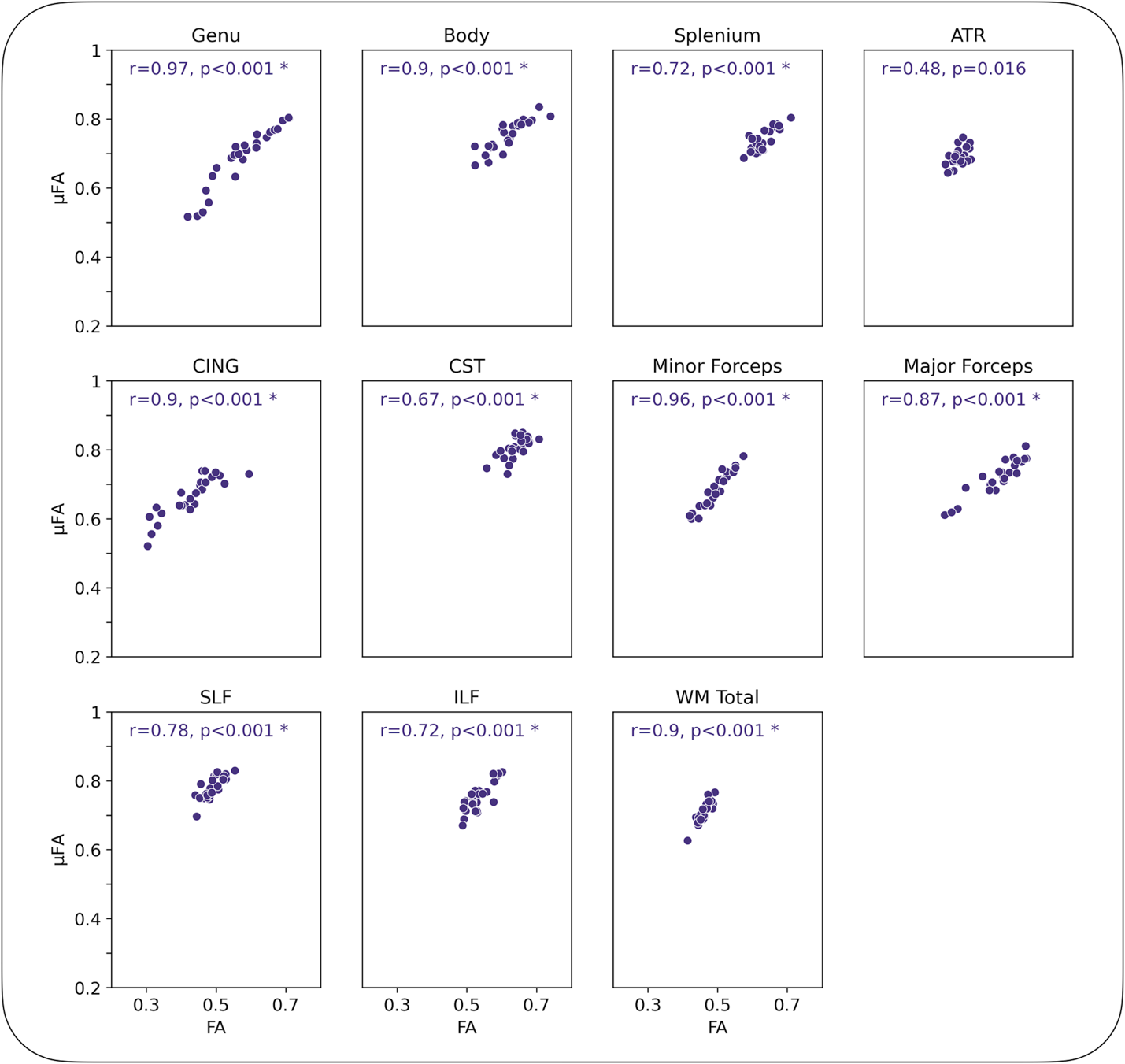


**Figure S6.** Comparison of FA and µFA separated by structure, with each point representing a subject’s metric value for that structure. The multiple comparisons were corrected using a Bonferroni correction (11 comparisons, resulting in a significance level of p < 0.004, and all relationships met this threshold). (*) indicates *p* < 0.004.


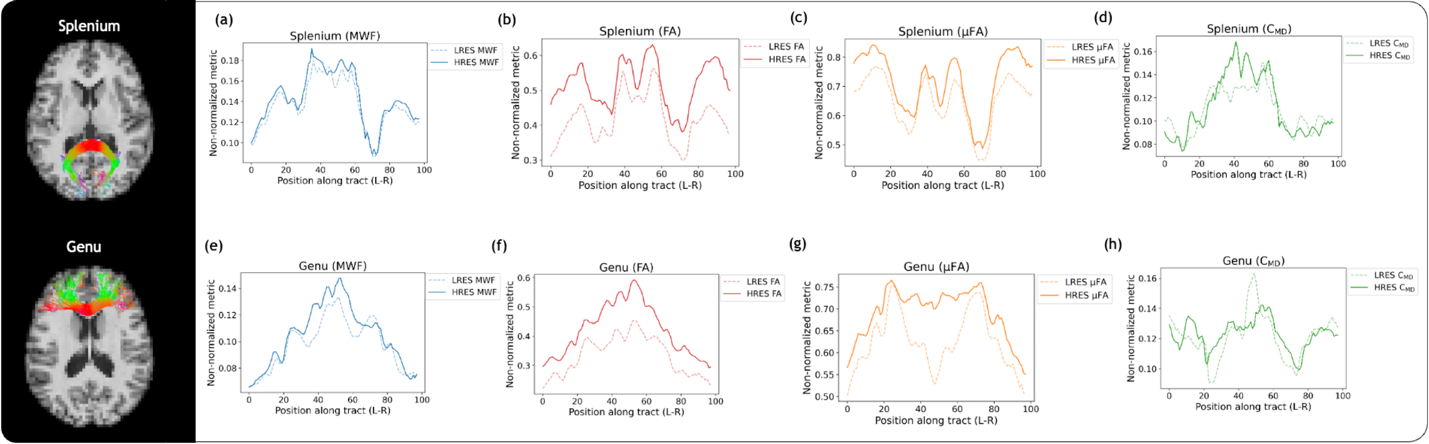


**Figure S7.** Original (3mm, Lower Resolution LRES, dashed line) and higher (2.25mm, Higher Resolution HRES, solid line) resolution image tract profiles in one healthy participant, separated by metric and presented without normalization for comparison. In the splenium, the main valley of µFA (c, position 40-60) is less dramatic in the HRES image than LRES although it is still present; C_MD_ shows a corresponding rise (d), while MWF stays relatively stable through position 40-60 (a). In the genu, the valley in µFA is less strong (g, position 40-60) in the HRES than the LRES while MWF stays similarly high through those positions. In both the genu and splenium, FA follows the same patterns at both resolutions. For both tracts, the HRES MWF and LRES MWF were derived from the same MWF dataset resampled to match the resolution of the tensor-valued diffusion data.
